# Hydrogen saline offers neuroprotection by reducing oxidative stress in a focal cerebral ischemia-reperfusion rat model

**DOI:** 10.1186/2045-9912-1-15

**Published:** 2011-07-05

**Authors:** Ying Liu, Wenwu Liu, Xuejun Sun, Runping Li, Qiang Sun, Jianmei Cai, Zhimin Kang, Shijun Lv, John H Zhang, Wei Zhang

**Affiliations:** 1 Department of Pathology, Weifang Medical College, Shandong, 261042, PR China; 2 Department of Diving Medicine, Second Military Medical University, Shanghai 200433, China; 3 Department of Neurology, Changhai Hospital,174 Changhai Road, Shanghai 200433, PR China; 4 Department of Physiology and Pharmacology, Loma Linda University School of Medicine, Loma Linda, California, USA

## Abstract

Hydrogen gas is neuroprotective in cerebral ischemia animal models. In this study, we tested the neuroprotective effects of hydrogen saline, which is safe and easy to use clinically, in a rat model of middle cerebral artery occlusion (MCAO). Sprague-Dawley male rats weighting 250-280 g were divided into sham, MCAO plus hydrogen saline and MCAO groups, and subjected to 90-min ischemia followed by 24 h of reperfusion. Hydrogen saline was injected intraperitoneally at 1 ml/100 g body weight. Infarct volume and brain water content were evaluated at different time points after reperfusion. Oxidative stress, inflammation, and apoptotic cell death markers were measured. Hydrogen saline significantly reduced the infarct volume and edema and improved the neurological function, when it was administered at 0, 3 and 6 h after reperfusion. Hydrogen saline decreased 8-hydroxyl-2'-deoxyguanosine (8-OHdG), reduced malondidehyde, interleukin-1β, tumor necrosis factor-α, and suppressed caspase 3 activity in the ischemic brain. These findings demonstrated hydrogen saline is neuroprotective when administered within 6 h after ischemia. Because hydrogen saline is safe and easy to use, it has clinical potentials to reduce neurological injuries.

## Introduction

Stroke is the second most frequent cause of death worldwide and the most frequent cause of permanent disability [1,2]. Advances in intravascular techniques and thrombolytic agents have reduced functional deficits within an optimal time window in stroke patients. However, reperfusion itself generates an over-production of reactive oxygen species (ROS), leading to reperfusion injury [3]. The burst of ROS is involved in the direct cytotoxic effects, including protein and lipid peroxidation, oxidative DNA damage, and post-ischemic inflammatory injury, through redox-mediated signaling pathways [4,5]. Therefore it is important to scavenge the free radicals and suppress the inflammation.

Hydrogen gas has been used in medical applications to prevent decompression sickness (DCS) in deep divers for safety profiles [6]. In 2007, Ohsawa et al found that molecular hydrogen can selectively reduce hydroxyl radical (OH) and peroxynitrite (ONOO^-^) in cell-free systems and exert a therapeutic antioxidant activity in rat middle cerebral artery occlusion (MCAO) model [7]. Some other observations showed that hydrogen also had the protective effect on ischemia-reperfusion injury in the intestine, liver and heart through the inhibition of oxidant stress [8-10].

Hydrogen gas would be much cheaper than other antioxidants if it could be clinically applied. However, hydrogen inhalation is not convenient and may be dangerous because it is inflammable and explosive if the concentration of hydrogen in the air is greater than 4%. On the other hand, after saturated in the physiological saline, molecule hydrogen in the saline is more easy to apply and safer than hydrogen inhalation. Considering the safety and the convenience, hydrogen saline has been prepared in our department and our previous experiments have demonstrated the neuroprotective effects of intraperitoneal hydrogen saline in a neonatal hypoxia-ischemia rat model [11]. Additionally, significantly improved post-ischemic functional recovery of rat hearts has also proved after hydrogen saline treatment [12]. The present study aimed to investigate the neuroprotective effect of hydrogen saline in the rat MCAO model.

## Materials and methods

### Experimental Protocol

All experimental procedures and protocols used in this study were reviewed and approved by the Animal Care and Use Committee of the Second Military Medical University. Furthermore, all were in accordance with the Guide for the Care and Use of Laboratory Animals. A total of 228 male Sprague-Dawley rats weighing 250-280 g were used in the present study. The rats were housed at 22-24°C under a 12-h-light/12-h-dark cycle, with food and water available *ad libitum *throughout the studies. Rats were randomly distributed into three groups, sham group (n = 52), MCAO group (n = 72) and MCAO plus hydrogen group (n = 104). Rats in the sham group only received intraperitoneal administration of normal saline and those in the MCAO group underwent MCAO followed by administration of normal saline at different time points (0, 3 or 6 h) after reperfusion onset. However, rats in the MCAO plus hydrogen group received MCAO and intraperitoneal treatment with hydrogen saline (1 ml/100 g body weight) at designed time points (0, 3 or 6 h after reperfusion onset). MCAO was produced by the filament model initially reported by Zea-Longa et al [13] with some modifications. After 90 min of right middle cerebral artery occlusion, the reperfusion of the MCA was initiated by removing the MCA occlusive filament. Rats were sacrificed at 12, 24, 72 h, and 7 days after reperfusion, and immunihistochemistry and detections of malondidehyd (MDA), anti-superoxide anion, interleukin-1β (IL-1β) and tumor necrosis factor-α (TNF-α) were performed.

### Neurological Scores

Neurological function was assessed using a standard scoring system [14]: 0 = no apparent deficits, 1 = contralateral forelimb flexion, 2 = decreased grip of contralateral forelimb, 3 = contralateral circling if pulled by tail, 4 = spontaneous contralateral circling.

### Evaluation of Infarct Volume

Infarct volume was determined by staining with 2, 3, 5-triphenyltetrazolium chloride (TTC, Sigma) as previously described [15]. The infarct and total hemispheric areas of each section, at intervals of 2-mm in thickness, were traced and analyzed using image analysis system (Image J software). The infarct ratio was calculated by dividing the infarct volume by the total volume of the sections.

### Brain Water Content

The brains were obtained and right hemisphere was quickly separated. Brain samples were weighted with a precise electronic balance and dried in an oven at 100 °C for 48 h [16]. Then, the samples were re-weighed and the water content was determined according to the following formula: [(wet weight - dry weight)/wet weight] × 100%.

### Nissl staining

For Nissl staining, the 4-μm sections were hydrated in 1% toluidine blue at 50 °C for 20 min. After rinsing with double distilled water, they were dehydrated and mounted with permount. The cortex from each animal was captured and Imaging-Pro-Plus (LEIKA DMLB) was used to perform quantitative analysis of cell numbers.

### Tunel staining

Terminal deoxynucleotidyl transferase dUTP nick end labeling (TUNEL) was performed on paraffin-embedded sections by using the *in situ *cell death detection kit (Roche). According to standard protocols, the sections were de-paraffinized and rehydrated by heating the slides at 60 °C. Then these sections were incubated in a 20 μg/ml proteinase K working solution for 15 min at room temperature. The slides were rinsed three times with phosphate buffer solution (PBS) before they were incubated in TUNEL reaction mixture for 1 h at 37 °C. Dried area around sample and added Converter-AP on samples for 1 h at 37 °C. After rinsing with PBS (5 min, 3 times), color development was performed in dark with nitroblue tetrazolium (NBT) and 5-bromo-4-chloro-3-indolylphosphate (BCIP).

### Immunohistochemistry

Immunohistochemistry was performed on 20 μm-thick free-floating coronal sections, which were prepared as previously described [17]. After incubation in 3% hydrogen peroxide (H_2_O_2_) in PBS, the sections were incubated overnight at 4 °C with primary antibodies against 8-hydroxyl-2'-deoxyguanosine (8-OHdG, 100:1; America Alpha Diagnostic international, a marker for DNA damage), Nitrotyrosine (40:1; America upstate, a marker for nitration), bax (100:1; America Abcam) and bcl-2 (600:1; Americ Millipore). Sections were then treated with secondary antibodies (1:2000, Vectastain, Vector Laboratories). Immunoreactivity was visualized subsequently by the avidin-biotin complex method (Vectastain, Vector Laboratories) as described previously [17].

### Cell counting

In each section, 6 visual fields (0.6 mm^2^) of cerebral cortex were randomly photographed. The number of staining cells in each field was counted at higher magnification (×200). Data were expressed as the number of cells per high-power field.

### Detection of MDA

Lipid peroxidation levels were measured with the thiobarbituric acid (TBA) reaction. This method was used to obtain a spectrophotometric measurement of the color produced during the reaction of TBA with MDA at 535 nm. For this purpose, 2.5 ml of 100 g/l trichloroacetic acid solution was added to 0.5 ml of homogenate in centrifuge tube followed by heating in boiling water for 15 min. The mixture was allowed to cool to room temperature and centrifuged (Eppendorf, 5810R) at 1000 × g for 10 min. Then, 2 ml of supernatant was added to 1 ml of 6.7 g/l TBA solution in a test tube, followed by heating in boiling water for 15 min. The solution was then cooled and the absorbance was measured with a spectrophotometer (UV-WFZ75, Shanghai, China). TBARS levels were expressed as nmol/mg protein in the brain.

### Caspase-3 activity assay

Brain samples from the cortex and hippocampus were taken from the impaired hemispheres of rats 24 h after hydrogen saline administration. The activity of caspase-3 was measured with caspase-3/CPP32 Fluorometric Assay Kit (BIOVISION Research Products 980, USA). Briefly, brain samples were homogenized in ice-cold lysis buffer and kept at 4 °C for 1 h. Brain homogenate was centrifuged at 12,000 g for 15 min at 4 °C. The supernatant was collected and stored at -80 °C for use. Protein concentration was measured using the Enhanced BCA Protein Assay Kit. A total of 50 μg of cell lysates were incubated in a 96-well plate with 2 × Reaction Buffer (50 μl). The reaction was started by adding 1 mM DEVD-APC substrate (5 μl). After incubation in dark at 37°C, the plate was read with a fluorometer equipped with a 400-nm excitation filter and 505-nm emission filter.

### Determination of IL-1β and TNF-α Levels

The levels of IL-1β and TNFα of brain tissues were determined with solid phase sandwich ELISA kit (Invitrogen, USA) under a microplate reader (Stat Fax 3200) at 450 nm.

## Results

### Infarct volume was reduced and brain edema was improved after treatment with hydrogen saline

**Figure 1:**
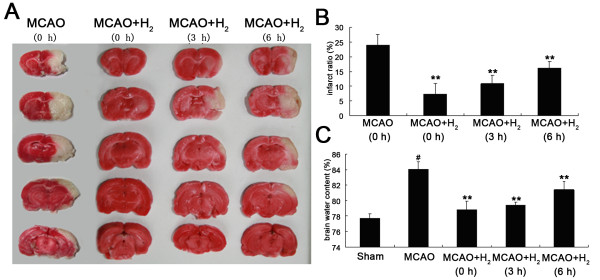
**Infarction and brain edema**. A: Coronal sections from ischemic rat brain stained with TTC. Administration of hydrogen saline was performed 0, 3, or 6 h after reperfusion. B: Hydrogen saline significantly reduced infarct volume when compared with MCAO group (**P < 0.01 vs. MCAO). C: Brain water content was increased at 24 h after MCAO (^#^*P *< 0.05 vs. sham) and hydrogen saline reduced brain edema when applied at 0, 3, or 6 h after reperfusion (**P < 0.01 vs. MCAO).

The hydrogen was administered 0, 3 and 6 after reperfusion to explore the preferable therapeutic regimen and brains were removed as 24 h after reperfusion followed by detection of infarction and brain edema. Hydrogen saline significantly reduced the infarct ratio when applied at 0 or 3 h after reperfusion, but that was only slightly reduced when injected at 6 h after reperfusion (Figure 1A-B). The mean brain water content of injured hemisphere was 77.67 ± 0.6% in the shame group, 84.04 ± 0.96% in the MCAO group and 78.82 ± 1.09% (0 h), 79.38 ± 0.44% (3 h), and 81.41 ± 1.02% (6 h) in the MCAO plus hydrogen group (*P *< 0.01) (Figure 1C). Based on these results, application of hydrogen 3 h after reperfusion was used in the following experiments.

### Body weight loss was decreased accompanied by improved neurological score after hydrogen saline treatment

**Figure 2:**
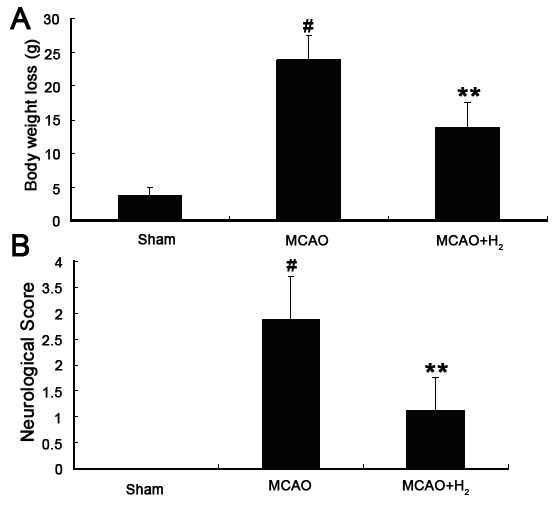
**Body weight and neurological function**. A. Body weight was markedly reduced at 24 h after MCAO when compared with pre-insult body weight and sham animals (^#^*P *< 0.05 vs. sham). Hydrogen saline treatment applied at 3 h after reperfusion partially prevented body weight loss (**P < 0.01 vs. MCAO). B. Neurological scores were significantly decreased (higher) after MCAO (^#^*P *< 0.05 vs. sham) and hydrogen saline applied at 3 h after reperfusion improved neurological function when compared with MCAO group (**P < 0.01).

The brain weight and neurological score were performed 24 after reperfusion. In the MCAO group, a marked body weight loss was observed when compared with sham group (*P *< 0.05). After hydrogen saline treatment at 3 h after reperfusion, the body weight loss was 23.93 ± 3.60 g in the MCAO group and 13.91 ± 3.64 g in the hydrogen treated group (*P *< 0.01), but marked body weight loss was also observed between the hydrogen treated group and the sham group (*P *< 0.05) (Figure 2A). Neurological scores were dramatically reduced in the MCAO group (*P *< 0.05 vs. sham), and hydrogen saline treatment at 3 h after reperfusion significantly improved the neurological function (*P *< 0.01 vs. MCAO group) even though neurological dysfunction was still observed (*P *< 0.05 vs. sham group) (Figure 2B).

### Nissl staining showed more viable cells after treatment with hydrogen saline

**Figure 3:**
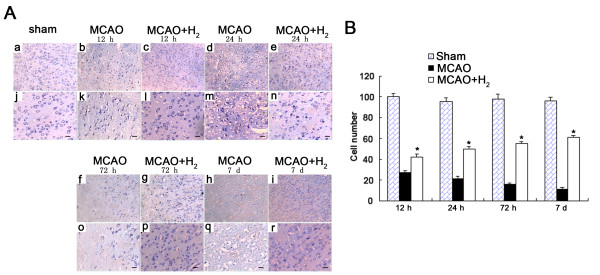
**Nissl staining and cell counting**. A: Nissl Staining was evaluated around the ischemic cortex. In all MCAO groups, shrunken neurons with intercellular space enlarged were observed and many unstained regions were observed. Hydrogen saline treated at 3 h after reperfusion preserved Nissl positive cells up to 7 days. B: The number of cells was significantly higher in hydrogen saline group (*P < 0.05 vs. MCAO). (a, b, c, d, e, f, g, h, i: 100 ×; j, k, l, m, n, o, p, q, r: 200 ×; Scale bar: 20 μm).

Figure 3A showed the Nissl staining of injured cortex at 12, 24, 72 h and 7 days after reperfusion. Numerous neuronal cells in the ischemic core died or shrunk with enlarged intercellular space. The cells were much better preserved in the hydrogen treated group and the number of viable cells was markedly increased (Figure 3B) (*P *< 0.05 vs. MCAO group).

### DNA oxidation was declined demonstrated by 8-OHdG staining after hydrogen saline treatment

**Figure 4:**
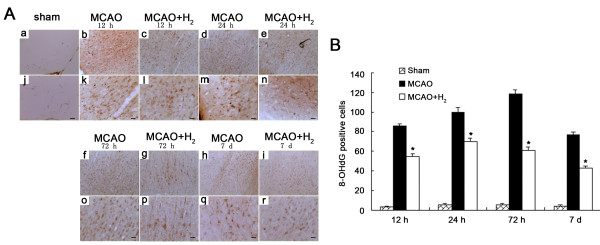
**8-OHdG staining**. A: No 8-OHdG positive staining was obtained in sham animals. Marked 8-OHdG positive staining was observed in the normal saline treated animals and the staining was localized to the nuclei of neurons. Hydrogen saline animals showed fewer number of 8-OHdG positive cells. B: Hydrogen saline applied at 3 h after reperfusion significantly reduced 8-OHdG positive cells when compared with MCAO group (**P < 0.01 vs. MCAO). (a, b, c, d, e, f, g, h, i: 100 ×; j, k, l, m, n, o, p, q, r: 200 ×; Scale bar: 20 μm).

Oxidative DNA damage was determined by measuring the amount of 8-OHdG in the injured cortex 12, 24, 72 h and 7 days after reperfusion. Less positive staining was detected in the sham operation group. After 12 h of reperfusion, strong 8-OHdG positive staining was observed in the nuclei of neurons located in the ischemic cortex in the MCAO group. Hydrogen saline treatment significantly decreased the number of 8-OHdG-positive cells (*P *< 0.05 vs. MCAO group) (Figure 4A, B).

### Number of apoptotic cells was decreased in TUNEL staining by hydrogen saline treatment

**Figure 5:**
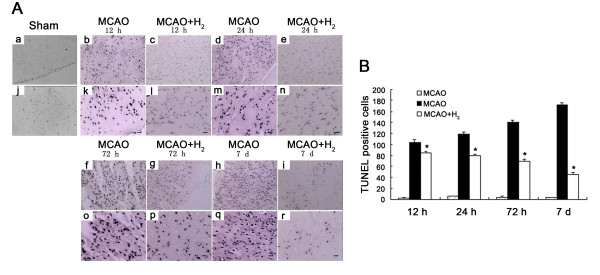
**TUNEL staining**. A: The TUNEL-positive material was localized in the nuclei of neurons as shown in the MCAO group. The damaged cells were characterized by a round and shrunken morphology. Fewer TUNEL positive cells were observed in hydrogen treated animals. B: Hydrogen saline applied 3 h after reperfusion markedly reduced the number of TUNEL positive cells when compared with normal saline treated group (*P < 0.05 vs MCAO). (a, b, c, d, e, f, g, h, i: 100 ×; j, k, l, m, n, o, p, q, r: 200 ×; Scale bar: 20 μm).

In the MCAO group, numerous TUNEL positive cells were observed and the injured cells were characterized by a round and shrunken morphology in the injured cortex at 12, 24, 72 h and 7 days after reperfusion. Hydrogen saline dramatically decreased the number of TUNEL-positive cells when applied at 3 h after reperfusion (*P *< 0.05) (Figure 5A, B).

### After hydrogen saline treatment, expressed of Bcl-2 was increased accompanied by decreased Bax expression and Capase-3 activity

**Figure 6:**
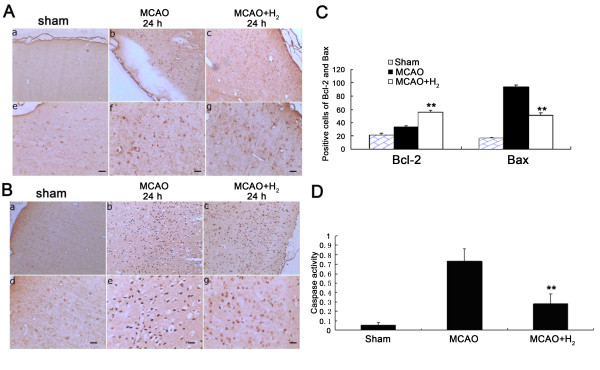
**Bcl-2, Bax and caspase-3**. A: Bcl-2 expression. Compared with sham animals, vehicle treated group showed a slight more positive staining of Bcl-2 cells. Hydrogen saline increased the number of Bcl-2 cells (Upper panel: 100 ×; Lower panel: 200 ×; Scale bar: 20 μm). B: Bax expression. A marked increase of Bax positive cells was observed in samples collected from vehicle treated animals, and hydrogen saline reduced the number of Bax positive cells (Upper panel: 100 ×; Lower panel: 200 ×; Scale bar: 20 μm). C. Hydrogen saline applied at 3 h after reperfusion increased the number of positive BCl-2 cells and reduced the number of positive Bas cells at 24 h after MCAO (**P < 0.01 vs. MCAO). D. A marked increase of caspase-3 activity was observed in the vehicle treated animals and hydrogen saline applied at 3 h after reperfusion decreased caspase-3 activity at 24 h after MCAO (**P < 0.01 vs. MCAO).

Results showed the number of cells positive for Bcl-2 or Bax in sham group was lower than that in MCAO group (P < 0.05). However, when compared with MCAO group, hydrogen saline applied 3 h after reperfusion significantly increased the expression of Bcl-2 (Figure 6A, C), and decreased that of Bax at 24 h after reperfusion (Figure 6B, C) (*P *< 0.01 vs. MCAO group). Similarly, when compared with sham group, the activity of caspase-3 was increased in the ischemic cortex of MCAO group at 24 h after reperfusion, which was significantly reduced by hydrogen saline treatment at 3 h after reperfusion (*P *< 0.01 vs. MCAO group) (Figure 6D) which however was still higher than that in the sham group (P < 0.05 vs. sham).

### Lipid peroxidation was improved by hydrogen saline treatment

**Figure 7:**
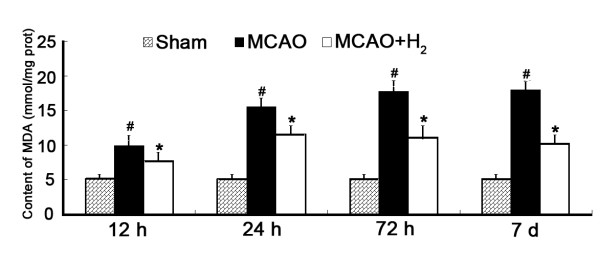
**MDA levels**. The content of MDA in the ischemic cortex was increased at 24 h after MCAO (^#^*P *< 0.05 vs. sham) and hydrogen saline significantly reduced MDA levels when compared with MCAO group (*P < 0.05 vs. MCAO).

The lipid peroxidation was presented as the MDA level at 12, 24, 72 h and 7 days after reperfusion. In the MCAO group, the MDA level in the ischemic cortex was increased which was significantly alleviated by hydrogen saline administration at 3 h after reperfusion (*P *< 0.05 vs. MCAO group) (Figure 7).

### Levels of IL-1β and TNF-α were decreased by hydrogen saline treatment

**Figure 8:**
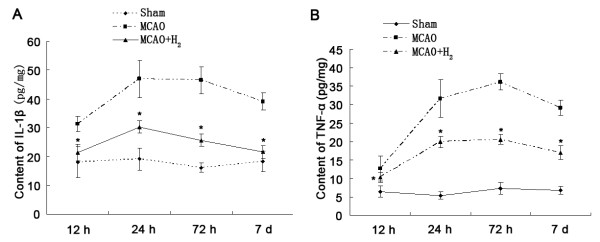
**IL-1β and TNF-α**. Protein levels of IL-1β (A) and TNF-α (B) were increased and lasted for 7 d after MCAO. Hydrogen saline applied at 3 h after reperfusion significantly reduced the levels of these inflammatory cytokines (*P < 0.05 vs. MCAO).

When compared with sham group, the levels of IL-1β and TNF-α were increased after MCAO and the increased levels of IL-1β and TNF-α lasted at least 7 days. After application of hydrogen saline at 3 h after reperfusion, the elevated levels of IL-1β and TNF-α were markedly decreased (*P *< 0.05 vs. MCAO group) (Figure 8).

## Discussion

In the present study, we evaluated the neuroprotective effects of hydrogen saline against cerebral ischemia-reperfusion injury. The major findings were that hydrogen saline could reduce cerebral infarction and improve neurological function in the MCAO rat model, which were mediated by the reduction of oxidative stress (8-OHdG, nitrotyrosine and MDA) and inflammatory factors, and subsequent decrease of neuronal apoptosis (TUENL positive cells, expression of Bcl-2 and Bax, and caspase-3 activity). The therapeutic window of hydrogen saline was similar to other prominent neuroprotectants. The protective effects were more pronounced if they were applied immediately after reperfusion, but the protective effects could be achieved to a certain extent when they were applied at 6 h after reperfusion. Our findings were consistent with previous studies in which protective effects of hydrogen gas through scavenging ROS have been confirmed in a cardiac ischemia-reperfusion injury model [12].

Increasing evidence has demonstrated ROS contribute to ischemia/reperfusion induced brain damage in a 2-phase pattern, an immediately occurring direct cytotoxic damage and a post-ischemia/reperfusion inflammatory injury [18]. ROS is massively produced in the brain after ischemia/reperfusion, and oxidative damage to brain tissues has been regarded as a fundamental mechanism of brain damage after transient or permanent cerebral ischemic injury [19,20]. All of these species interact with nearby cellular components, such as proteins, lipids, and DNA [4,5]. Some components in the reactive oxygen species such as superoxide anion and H_2_O_2 _can be detoxified by antioxidant defense enzymes, while there is no enzyme to detoxify OH and ONOO^-^, extremely reactive free radicals in cells, until a recent study reported that hydrogen gas could selectively reduce these two harmful free radicals [7]. Hydrogen molecule is electronically neutral and has the ability to penetrate the membranes of cell, nucleus and mitochondria. 8-OHdG is a product of direct oxidation of DNA by hydroxyl radicals and has been used as a marker for oxidative stress [21]. Our results showed reduced number of 8-OHdG positive cells after MCAO by hydrogen saline. Our findings were consistent with a recent study on hydrogen inhalation in which hydrogen inhalation also reduced the oxidative stress following ischemia/reperfusion [22].

Oxidative stress can also lead to inflammatory response after ischemic stroke, which is characterized by enhanced cytokines production [23]. Among the known cytokines, IL-1β and TNF-α are produced by macrophages, endothelial cells, astrocytes and neurons, and play crucial roles in the ischemic brain injury [23]. Reduction of oxidative stress by hydrogen saline may result in the suppressed production of TNF-α and IL-1β as demonstrated by our study. A possible direct anti-inflammatory effect of hydrogen saline in cerebral ischemia warrants further investigation.

Oxidative stress and inflammation contribute to the activation of program cell death following cerebral ischemia [24]. Oxidative stress can cause changes in the mitochondrial permeability resulting in the release of cytochrome c which then activates caspase-3 executing cell death signals. By reducing oxidative stress and inflammation, hydrogen saline suppressed caspase-3 activity in the ischemic cortex, which might be related to the decreased release of cytochrome c. Two other important mitochondrial apoptotic factors Bcl-2 and Bax were examined in the present study [25]. Consistently, hydrogen saline treatment also up-regulated the Bcl-2 expression and down-regulated the Bax expression.

Of note, although the protective effects were also observed in our previous study, the therapeutic effects of hydrogen saline were more profound than those of hydrogen inhalation. In addition, the effects of intravenous administration of hydrogen were inferior to those of intraperitoneal treatment, which may be explained by rapid elimination of hydrogen through pulmonary gas exchange. But the exact mechanism should be further investigated. Although the intravenous application was more clinical than intraperitoneal administration, intraperitoneal injection was frequently performed in animals. Therefore, in the present study, intraperitoneal administration of hydrogen saline was conducted to observe the neuroprotective effects. Furthermore, in our pilot study on animals and humans, some parameters did not show evident side effects even with several large doses of hydrogen saline were applied.

Taking together, hydrogen has been shown anti-oxidative stress and is beneficial on lipid and glucose metabolism in humans [26]. Hydrogen water also decreased superoxide formation caused by ischemia-reperfusion in the brain slices of mice [27]. For the safety and the convenience of hydrogen administration, hydrogen saline was prepared and protective effects of hydrogen saline confirmed in rat cardiac ischemia/reperfusion and neonatal hypoxia-ischemia models [11,12]. In the present study, we further demonstrated that intraperitoneal administration of hydrogen saline yielded similar neuroprotective effects comparable to hydrogen inhalation [7,22]. Therefore, our study for the first time showed hydrogen saline had potentials as an alternative pharmacological strategy in ischemic stroke.

## Competing interests

The authors declare that they have no competing interests.

## Authors' contributions

LY and LWW contributed equally to this work. LY carried out the molecular studies and drafted the manuscript; LWW participated in the design of the study, revised the paper and performed the statistical analysis; LRP, SQ, CJM and LSJ participated in its design and coordination; KZM performed the histological examination; ZJH revised this paper and participated in coordination, ZW and SX conceived of the study and participated in the design of the study. All authors read and approved the final manuscript.
